# Efficacy and safety of exogenous ketone bodies for preventive treatment of migraine: A study protocol for a single-centred, randomised, placebo-controlled, double-blind crossover trial

**DOI:** 10.1186/s13063-018-3120-7

**Published:** 2019-01-17

**Authors:** Elena Gross, Niveditha Putananickal, Anna-Lena Orsini, Simone Schmidt, Deborah R. Vogt, Sven Cichon, Peter Sandor, Dirk Fischer

**Affiliations:** 1 Division of Neuropaediatrics, University of Basel Children’s Hospital, University of Basel, Spitalstrasse 33, Postfach, 4056 Basel, Switzerland; 2 Department of Clinical Research, Clinical Trial Unit, University of Basel Hospital, University of Basel, Basel, Switzerland; 3 Department of Medical Genetics, University of Basel Hospital, University of Basel, Basel, Switzerland; 4 RehaClinic, Bad Zurzach, Switzerland

**Keywords:** Migraine, Migraine prevention, Exogenous ketone bodies, Beta-hydroxybutyrate, 3-Hydroxybutyrate, Ketosis, Randomised controlled trial, Placebo-controlled, Crossover, Clinical trial

## Abstract

**Background:**

Currently available prophylactic migraine treatment options are limited and are associated with many, often intolerable, side-effects. Various lines of research suggest that abnormalities in energy metabolism are likely to be part of migraine pathophysiology. Previously, a ketogenic diet (KD) has been reported to lead to a drastic reduction in migraine frequency. An alternative method to a strict KD is inducing a mild nutritional ketosis (0.4–2 mmol/l) with exogenous ketogenic substances. The aim of this randomised, placebo-controlled, double-blind, crossover, single-centre trial is to demonstrate safety and superiority of beta-hydroxybutyrate (βHB) in mineral salt form over placebo in migraine prevention.

**Methods/design:**

Forty-five episodic migraineurs (5–14 migraine days/months), with or without aura, aged between 18 and 65 years, will be recruited at headache clinics in Switzerland, Germany and Austria and via Internet announcements. After a 4-week baseline period, patients will be randomly allocated to one of the two trial arms and receive either the βHB mineral salt or placebo for 12 weeks. This will be followed by a 4-week wash-out period, a subsequent second baseline period and, finally, another 12-week intervention with the alternative treatment. Co-medication with triptans (10 days per months) or analgesics (14 days per months) is permitted. The primary outcome is the mean change from baseline in the number of migraine days (meeting International Classification of Headache Disorders version 3 criteria) during the last 4 weeks of intervention compared to placebo. Secondary endpoints include mean changes in headache days of any severity, acute migraine medication use, migraine intensity and migraine and headache-related disability. Exploratory outcomes are (in addition to routine laboratory analysis) genetic profiling and expression analysis, oxidative and nitrosative stress, as well as serum cytokine analysis, and blood βHB and glucose analysis (pharmacokinetics).

**Discussion:**

A crossover design was chosen as it greatly improves statistical power and participation rates, without increasing costs. To our knowledge this is the first RCT using βHB salts worldwide. If proven effective and safe, βHB might not only offer a new prophylactic treatment option for migraine patients, but might additionally pave the way for clinical trials assessing its use in related diseases.

**Trial registration:**

ClinicalTrials.gov, NCT03132233. Registered on 27 April 2017.

**Electronic supplementary material:**

The online version of this article (10.1186/s13063-018-3120-7) contains supplementary material, which is available to authorized users.

## Background

Migraine is a complex, common and debilitating neurological disorder [1] that affects approximately 17% of women and 8% of men in Europe [2]. With a peak incidence during the most productive years of life, migraine not only causes a huge amount of suffering, but also inflicts a substantial amount of costs on society: approximately €18.5 billion per year in Europe alone [3].

Various lines of research suggest that brain energy metabolism abnormalities are likely to be part of migraine pathophysiology [4–9]. Specifically, there is some evidence for reversible abnormalities in mitochondrial functioning in migraine [7, 8, 10]. For example, treatment with riboflavin and coenzyme Q10 has been shown to have migraine-protective effects [4, 7, 9–12], probably via a positive effect on energy metabolism [7, 10]. Lactic and pyruvic acid, markers of mitochondrial disease, have been found to be increased in migraineurs [13]; ^31^P-MRS patterns seen in migraine are consistent with what is seen in mitochondrial disorders [5, 14–16]; and COX-negative fibres typical of mitochondrial diseases have also been seen in some patients with migraine [6]. A breakdown of the resting membrane potential due to lack of ATP could explain cortical abnormalities in excitability, which have been reported in migraine [17–21] and would offer a mechanism by which the trigeminal pain pathway, whose afferents densely innervate the meninges and its associated blood vessels, could be activated or sensitised in migraine. The activation and sensitisation of the trigeminal pain pathway is considered the current understanding for the origin of the migraine headache [22–24].

Despite causing a huge amount of suffering and a substantial amount of costs for society [3, 25], current migraine treatment options are limited and their mechanisms of action are also not completely understood [26]. Most of the prophylactic agents licensed to date are not migraine specific and are additionally associated with significant, sometimes intolerable, side-effects. Furthermore, their migraine-preventive properties tend to be moderate at most. Hence, there is a need for developing alternative anti-migraine therapies.

The ketogenic diet (KD) was developed about 100 years ago after the observation that prolonged fasting has anticonvulsive properties [27]. With its high fat, low carbohydrate and medium protein content, the KD simulates the metabolic effects of starvation. With the advent of antiepileptic medication the rather complicated KD fell out of favour. However, within recent years it has received new interest, in particular since ketone bodies (KBs) might be beneficial for a variety of neurological and even psychiatric disorders due to various different mechanisms [28–30], including improved energy metabolism.

Recently, some case studies [31–34] and a first short proof-of-concept study [35] have demonstrated a reduction in migraine attack frequency, severity and use of acute anti-migraine medication during ketosis—with effect sizes ranging from total absence of attacks [31] to a reduction to 1/5th of the run-in period [35]. In addition, preliminary evidence suggests that the migraine-protective effect may outlast the duration of ketosis [31, 32, 35]. This might be a result of longer-lasting gene expression changes [28, 36]. Elevated KB levels in humans have been shown to be well tolerated for extended periods of time (up to several years) [34, 37–48]. However, a strict KD might not provide a feasible long-term solution for all episodic migraine patients, because patient adherence may be limited and it is not easily implemented in an ambulatory setting.

An alternative means to induce a state of mild to medium nutritional ketosis (0.4–2 mmol/l), irrespective of blood glucose levels, is dietary supplementation with ketogenic substances, such as beta-hydroxybutyrate (βHB) salts [45, 49–52]. This approach could be easily implemented with intake of a ketogenic powder dissolved in water (consisting of a calcium–magnesium–βHB salt three times a day). This intervention seems much more feasible than a strict KD in larger patient populations and avoids the complications of a very restricted high-fat diet. These considerations led us to examine the efficacy and safety of KB mineral salts in migraine prevention within the scopes of a double-blind, randomised, placebo-controlled, efficacy and safety trial with a crossover design.

## Material and methods

### Study design and setting

**Fig. 1:**
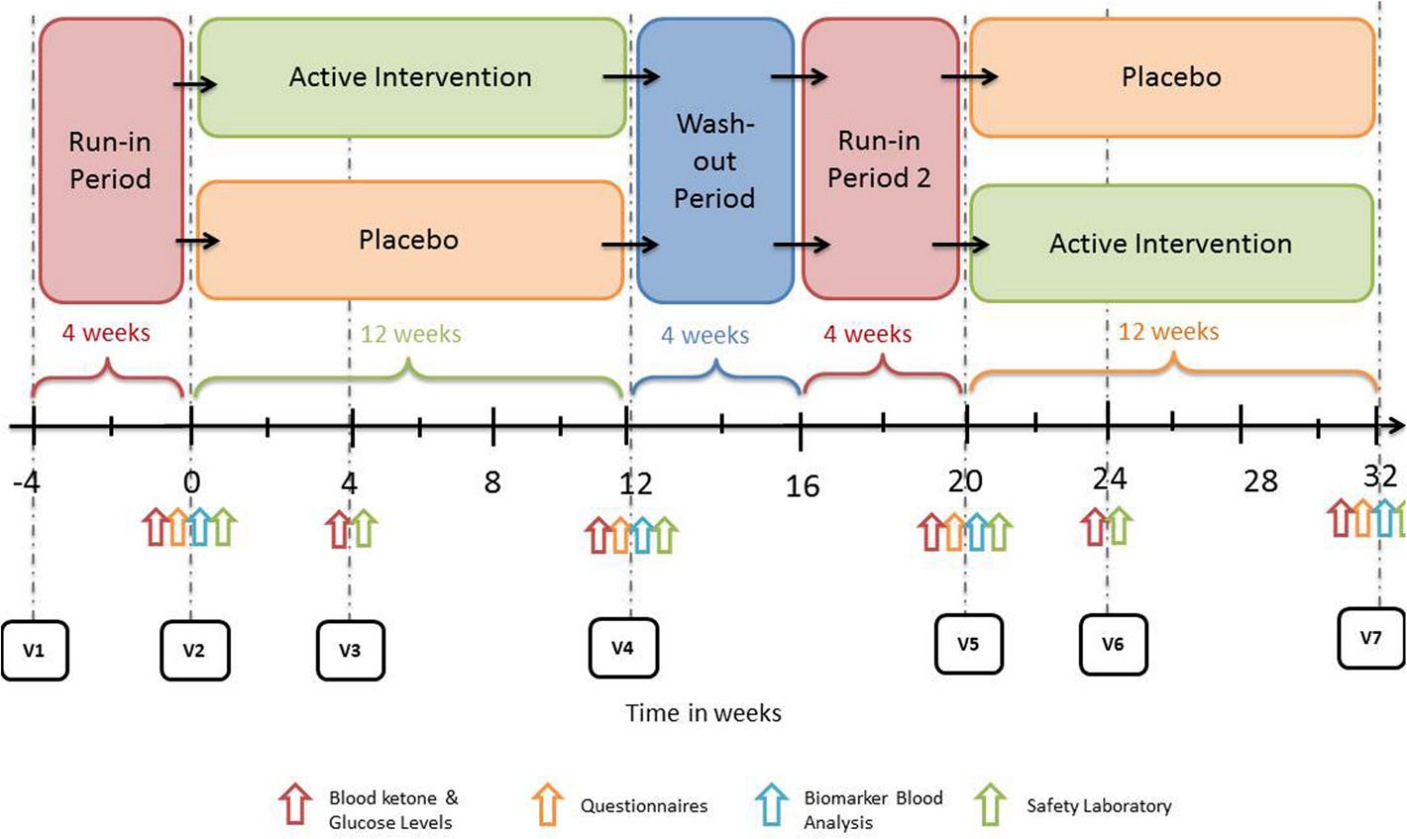
Flowchart of study design, including timing of measurements and procedures. V = visit

The study is an investigator-initiated, double-blind, randomised, placebo-controlled, efficacy and safety trial with a crossover design (see Fig. 1) and a treatment period of 36 weeks. It is a single-centre study; all investigations will take place at the clinical trial unit (CTU) of the University Hospital Basel (USB), Switzerland.

We plan to enrol 45 medium to high-frequency episodic migraineurs (5–14 migraine days/months), with or without aura, aged between 18 and 65 years.

The study period will begin with a 4-week run-in period, during which there is no investigational treatment. The purpose of the run-in period will be observation for baseline comparison. The run-in period will be followed by a 12-week intervention period, when the subjects will receive the investigational medicinal product (IMP) or placebo (orally, three times a day). The intervention period will be followed by a 4-week wash-out and a 4-week second run-in period, during which the subjects will receive no further intervention.

As the study medicament has a half-life of less than 4 h and the outcome measures are based on the last 4 weeks of the 12 weeks intervention only, a 4-week wash-out period was judged to be sufficient. This will be followed by a second 12-week intervention period of the alternative treatment (patients who first received placebo will now receive IMP and vice versa).

Ethics approval has been obtained from the local Ethics Committee (EKNZ 2015-304) and the corresponding competent authority (CA): the National Swiss Drug Agency (2016DR2109). The trial was registered at ClinicalTrials.gov (NCT03132233) prior to starting recruitment. Funding for the study has been received from the Swiss National Science Foundation (SNSF).

### Eligibility criteria

#### Inclusion criteria

##### Visit 1 (prior to the 4-week run in period)

The patient:

1. is between the ages of 18 and 65 years;
2. has been previously diagnosed with migraine (with or without aura) in accordance with the International Classification of Headache Disorders version 3 (ICHD-3) Beta Classification criteria;
3. experiences between 5 and 14 migraine days per month (over the last 4 months) with at least two of the migraines lasting more than 4 h;
4. has an age of onset of migraine younger than 50 years;
5. agrees to refrain from initiating or changing the type, dosage or frequency of any prophylactic medications (exclusive of medications taken for acute relief of migraine symptoms) as well as dietary supplements (such as Q10, riboflavin, etc.) against migraine and for indications other than migraine that in the opinion of the clinician may interfere with the study objectives (e.g. antidepressant, anticonvulsants, beta blockers, etc.) for the duration of the study;
6. has not changed type, dosage or frequency of any prophylactic medications (exclusive of medications taken for acute relief of migraine symptoms) as well as dietary supplements (such as Q10, riboflavin, etc.) against migraine and for indications other than migraine that in the opinion of the clinician may interfere with the study objectives (e.g. antidepressant, anticonvulsants, beta blockers, etc.) for at least 3 months prior to study onset;
7. refrains from making any drastic changes to their diet for the duration of the study, including periods of fasting;
8. agrees to use the study medication as intended, follow all of the requirements of the study including follow-up visit requirements, record required study data in the subject dairy and other self-assessment questionnaires, and is okay with drawing blood samples; and
9. is able to provide written informed consent.

##### Visit 2 (baseline visit, just prior to 12-week intervention)

Before starting the intervention, the study patient must meet all of the following inclusion criteria.

The patient:

1. continues to meet all baseline (Visit 1) eligibility criteria;
2. has experienced between 5 and 14 migraine days; and
3. has demonstrated compliance with the headache diary during the run-in period.

#### Exclusion criteria

##### Visit 1 (prior to the 4-week run-in period)

Subjects meeting any of the following criteria cannot be included in this research study.

The patient:

1. has a concomitant medical condition that will require oral or injectable steroids during the study;
2. has a history of any significant neurological, psychiatric or other medical condition that in the opinion of the investigator may confound the study assessments (no liver or kidney diseases in particular);
3. has a cardiovascular disease (hypertension in particular) or a history thereof;
4. has a known history of suspected secondary headache;
5. currently takes simple analgesics or non-steroidal anti-inflammatory drugs (NSAIDs) for more than 14 days per 4 weeks or triptans for more than 10 days per 4 weeks for headaches or other body pain;
6. currently takes prescription opioids;
7. has previous diagnosis of medication overuse headache (MoH), which has reverted to episodic migraine within the last 6 months;
8. meets the ICHD-3 Beta Classification criteria for chronic migraine (> 15 headache days per month);
9. has failed an adequate trial (2 months or longer) of at least three classes of a drug therapy for the prophylaxis of migraine;
10. has had surgery for migraine prevention;
11. has received Botox injections within the last 6 months;
12. is pregnant or thinking of becoming pregnant during the study period, or is of childbearing years and unwilling to use an accepted form of birth control;
13. is participating in any other therapeutic clinical investigation or has participated in a clinical trial in the preceding 30 days;
14. belongs to a vulnerable population or has any condition such that his or her ability to provide informed consent, comply with the follow-up requirements or provide self-assessments is compromised (e.g. homeless, developmentally disabled or prisoner); and
15. is thinking to start, change or stop a hormone-based contraception.

##### Visit 2 (baseline visit, just prior to 12-week intervention)

Before starting the intervention, the study patient must meet none of the following exclusion criteria.

The patient:

1. has initiated or changed the type, dose or frequency of any prophylactic medication for indications other than migraine that in the opinion of the clinician may interfere with the study objectives during the 4-week run-in period

### Interventions

#### Experimental intervention

The investigational medicinal product (IMP) used in this clinical trial is d-l-beta-hydroxybutyrate (βHB) in powdered calcium (Ca^2+^)–magnesium (Mg^2+^)–salt form (Ca-Mg-βHB). d-l-Beta-hydroxybutyrate calcium salt (Ca-βHB) dissolves in water (i.e. in the body) into Ca^2+^ and d-l-beta-hydroxybutyrate (βHB), the compound of interest. d-l-Beta-hydroxybutyrate magnesium salt trihydrate (Mg-βHB) dissolves in water (i.e. in the body) into Mg^2+^ and βHB. Also known as beta-hydroxybutyric acid, 3-hydroxybutyric acid or 3-hydroxybutyrate, βHB is an endogenous metabolite with the formula CH_3_CH(OH)CH_2_CO_2_H. It is a beta-hydroxy acid and a keto acid. The IMP was purchased from Ergomax (https://www.ergomaxsupplements.com) in bulk powder of GMP quality and packaged at Hänseler AG (Herisau, Switzerland). It does not contain anything else other than the βHB mineral salts. The flavour is masked using a sucralose-based sugar-free syrup. The daily dose of 9 g Ca-βHB contains 7.54 g of βHB and 1.47 g Ca^2+^, and will be divided into three servings supplied in individual sachets containing 2.51 g βHB and 0.49 g Ca^2+^, respectively. The daily dose of 9 g Mg-βHB contains 6.6 g βHB and 0.77 g Mg^2+^, and will also be divided into three servings supplied in individual sachets containing 2.2 g βHB and 0.26 g Mg^2+^. Both IMPs are provided as a water-soluble powder. During the 12-week intervention period participants will consume the IMPs in three oral doses, to be taken with or after breakfast, lunch and dinner, respectively. This adds up to less than 100 kcal per day. Each serving will raise KB levels for approximately 3 h. To minimise possible gastrointestinal symptoms such as bloating or diarrhoea, patients are instructed to increase the dosage over time, starting with half the dose during the first week before reaching the maximum dosage by day 7.

Elevated ketone body (KB) levels have been shown to be well tolerated for extended periods of time (up to several years) [31–35, 37–49]. During fasting, the healthy adult is capable of producing up to 185 g of KBs [53]. Previously, orally administered sodium βHB salts with higher doses ranging between 0.5 and 1 g per kg have been shown to be tolerated in both the short term [39, 50, 54, 55] and the long term [45, 49, 52, 56, 57] with no significant side-effects. The rather conservative dose of 18 g βHB mineral salt per day (as compared to endogenous production during starvation) was determined largely by the mineral load of Mg^2+^ and Ca^2+^, which we wanted to keep within acceptable ranges. Not going over the suggested maximum supplemental guidelines meant 9 g of Mg-βHB and 9 g of Ca-βHB, respectively. A similar dose of 5 g βHB/day was shown to lead to a modest elevation in blood KB (up to 0.4 mmol/l) [52], supporting the safety of our chosen dose. A Ca-βHB and Mg-βHB salt was chosen to avoid the potentially negative long-term consequences of high sodium intake.

To the best of our knowledge, no human controlled trials using βHB mineral salts have been done, either for migraine or for any other indication, and there seems to not yet be other human published data on specifically Ca-βHB and Mg-βHB. However, recently, βHB supplements, mostly in mineral salt form similar to our IMP, are being produced and sold in the USA, marketed as a sport/life-style supplement. A couple of million servings with a similar dosing to our IMP have been consumed without any incidents reported.

#### Control intervention

The placebo powder consists of mannitol, a sugar alcohol, which has the same texture, colour and packaging. Taste and smell are masked in the applied form (both are diluted in sugar-free syrup) and therefore similar. It is used by the USB Pharmacy as the standard placebo substance. In higher doses it can also lead to gastrointestinal symptoms [58], which means it has similar potential side-effects to the IMP.

#### Packaging, labelling and supply

The IMP and placebo are provided in sachets containing either one dose of Ca-βHB (3 g) or one dose of Mg-βHB (3 g), respectively, in powder form (see earlier). The whole supply for the study (ca. 3 kg per patient, > approximately 70 kg IMP and 70 kg placebo in total) will be delivered to and stored at the pharmacy of the University Hospital Basel. Patients will be provided with sufficient quantity to last from each visit to the next. The IMP will be labelled in accordance with regulatory requirements.

#### Storage conditions

The IMP is stored at room temperature. After delivery it will be stored at the USB Pharmacy until the end of the study.

#### Concomitant interventions (treatments)

The use of analgesics and triptans is allowed for less than either 14 days (analgesics) or 10 days (triptans), respectively, per month. They are not predicated to have an effect on the study outcomes. Steroids (oral or injectable) as well as prescription opioids are not permitted for the duration of the study period, including run-in and follow-up. Migraine-related surgery and Botox injections within the last 6 months are also not permitted. Prophylactic medications (exclusive of medications taken for acute relief of migraine symptoms) as well as dietary supplements (such as CoenzymeQ10, riboflavin, etc.) against migraine and for indications other than migraine that in the opinion of the clinician may interfere with the study objectives (e.g. antidepressant, anticonvulsants, beta blockers, etc.) are permitted as long as the type, dosage or frequency is not changed for the duration of the study and has not been changed at least 3 months prior to study onset. Hormone-based contraception is permitted as long as the patient does not intend to start, stop or change it for the duration of the study and at least 3 months prior to the intervention. Other hormonal treatment is not permitted.

### Outcome measures

#### Primary outcome measure

##### Mean change from baseline in number of migraine days (meeting ICHD-3 criteria) during the last 4 weeks of intervention compared to placebo

In order to assess the therapeutic efficacy of externally induced mild ketosis over placebo in migraine prevention, a detailed headache diary in pen and paper form is used to record the change in monthly migraine frequency. The headache diary includes: month, days 1–31, distinction migraine/headache, pain intensity (Likert scale 0–10), medication, dosage, treatment effectiveness of acute medication used (Likert scale 0–10), migraine-associated symptoms, days with menstruation and potential trigger factors (if known).

A day with head pain will only be classified as a migraine day if it meets ICHD-3 classification criteria. According to the International Headache Association and the European Medical Association Guidelines, the recommended measure to assess migraine frequency reduction is the change in migraine days per 4 weeks compared to baseline. This approach has one major advantage over the other frequently used method of recording the number migraine attacks: attack duration is also taken into consideration.

#### Secondary outcome measures

##### Mean change from baseline in number of headache days of any severity (meeting ICHD-3 criteria) during the last 4 weeks of intervention compared to placebo

The same headache diary in pen and paper form is used to record the change in 4-week headache frequency. A day with headache will only be classified as a headache day if it does not meet ICHD-3 migraine classification criteria. According to the International Headache Association and the European Medical Association Guidelines, the change in migraine days versus headache days per 4 weeks compared to baseline should be recorded separately in migraine patients who experience both headache types.

##### Mean change from baseline in consumption of acute migraine medication (analgesics or triptans) measured in days with acute headache medication use during the last 4 weeks of the intervention

The same headache diary in pen and paper form is used to record the change in days with acute headache medication use (analgesics or triptans). With this approach the number of tablets is not of primary interest, but rather the number of days on which one or more analgesics or triptans were consumed. A clinically meaningful migraine preventative is predicted to lower the days during which migraine acute medication is necessary.

##### Mean change from baseline in migraine intensity (measured with a numerical rating scale from 1 to 10) during the last of 4 weeks of the intervention period

The same headache diary in pen and paper form is used to record a potential change in migraine intensity, as measured with a numerical rating scale from 1 to 10. Each migraine or headache day, respectively, is given an intensity score, with 0 being not painful at all and 10 being an operation without anaesthesia.

##### Change in disability from baseline during any treatment period, as assessed with the Migraine Disability Assessment and the Headache Impact Test (comparison baseline and post-intervention score)

In order to assess a change in migraine and headache-related disability, two commonly used validated and reliable questionnaires are used: the Migraine Disability Assessment (MIDAS) and the Headache Impact Test (HIT-6) [59–63]. The German translations were also shown to have adequate reliability and validity [60, 63]. Both questionnaires will be provided as pen and paper versions and will be filled out at the baseline visits (V2 and V5) and the end of innervation visits (V4 and V7), respectively.

#### Exploratory outcome measures

The demographic characteristics and neurological examination will be assessed at one time point (Visit 1). To determine the potential mechanisms of action of successful migraine treatment, we are going to examine single nucleotide polymorphism (SNP) markers in order to assess the genetic background of migraine patients involved in this study. In addition to this, we also plan to conduct gene expression analysis. SNP and gene expression analysis will be conducted using microarrays. In our analysis strategy we especially focus on, but not limit to, genes coding for mitochondrial-related enzymes (citrate synthase, cytochrome C oxidase subunit 1, succinate dehydrogenase subunit A).

We will also examine the serum concentration of oxidative and nitrosative stress markers (malondialdehyde (MDA), carbonylated proteins, nitrate, nitrite, nitrotyrosine) using enzyme-linked immunosorbent assay (ELISA) and mass spectroscopy. In addition to Hba1c, insulin, cortisol, lactate and markers of functioning, cytokines will be analysed using the MILLIPLEX MAP Human Cytokine/Chemokine Magnetic Bead Panel—Premixed 41 Plex—Immunology Multiplex Assay.

Optionally, patients will also receive an Abbott FreeStyle Libre Blood Glucose Monitoring System for 2 weeks at visits V2, V3, V5 and V6, respectively, which will allow permanent tissue glucose monitoring without finger pricking. This allows us to examine a potential association between blood glucose levels (hyperglycaemia or hypoglycaemia) and migraine, and the potential effect of the study medication on glucose levels.

### Safety outcomes measures

Safety and tolerability will be determined by:

1. comparison of treatment-emergent adverse events (any event regardless of potential causality with the drug) and treatment-related adverse events (such as gastrointestinal upset) as imputed by the principal investigator between active treatment and placebo; and
2. examination for potential effects of the intervention on routine laboratory parameters (renal and liver function tests, electrolytes, full blood count, lipids, glucose, CRP, Hba1c, insulin, cortisol, lactate, TSH, FT4 and FT3) in the treatment group compared to the control group.

### Study procedure

**Fig. 2:**
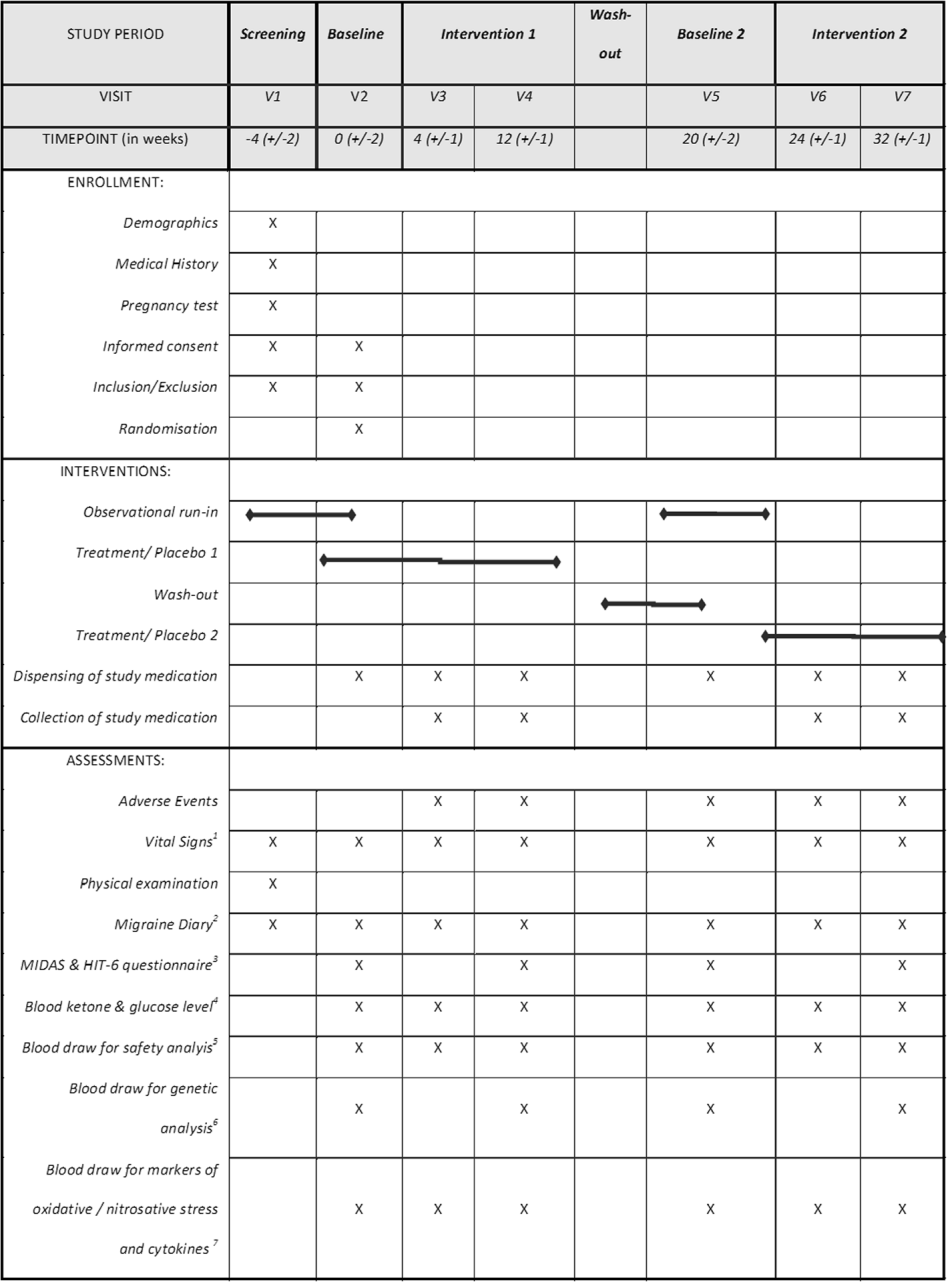
Detailed study schedule. ^1^Blood pressure, heart rate, weight and height. ^2^Pen and paper headache dairy. ^3^Migraine Disability Questionnaire (Migraine Disability Assessment (MIDAS)) and Headache Impact Test (HIT), German versions, standard questionnaires for assessing the extent of migraine-related disability. ^4^Blood beta-hydroxybutyrate and glucose levels, measured with a portable ketone meter (precision xtra by Abbot). ^5^Routine laboratory (renal and liver function tests, electrolytes, full blood count, C-reactive protein, serum cholesterol, triglycerides, serum proteins, albumin, glucose, Hba1c, insulin, cortisol, lactate, TSH, FT4 and FT4). ^6^Blood draw (1 × EDTA, 1 × PAXgene) at each time point for genetic profiling and gene expression analysis using microarrays. ^7^Blood draw at each time point for oxidative and nitrosative stress markers (malondialdehyde (MDA), carbonylated proteins, nitrite, nitrotyrosine) and serum cytokine measurements (including, but not limited to, IFN-γ, IL-1β, IL-2, IL-4, IL-5, IL-6, IL-10, MCP-1, TNF-α, TNF-β, TGF-β1). V = visit

At screening (Visit 1 (V1), week − 4), patients are informed about preclinical data, alternative treatments, potential risks and benefits of the study (see Fig. 2). Further, written informed consent, including consent for the collection of blood for genetic analysis, from the patients is obtained by the trial physician. After signing the informed consent form, the inclusion and exclusion criteria are verified. If the criteria are fulfilled, the patient will be enrolled in the study under reserve. During V1 the following additional procedures are performed: a detailed first clinical interview/examination, vital signs, migraine diary explanation, where necessary a pregnancy test and a neurological examination. After screening, visits will be scheduled for baseline (V2, week 0). V1 will last approximately 30 min.

At the start of the intervention (baseline/V2, week 0) the following procedures are performed: check inclusion/exclusion criteria and, if met, confirmation of enrolment, migraine diary check, diet check, consumption of first dose of IMP/placebo, adverse events, vital signs, physical examination if necessary, blood draw (for safety, biomarkers and genetic analysis), standardised migraine questionnaires, KB and glucose concentration measurements using a portable point-of-care blood ketone meter (precision xtra from Abbot) and/or the Abbott FreeStyle Libre Blood Glucose Monitoring System. Patients will be randomly assigned to the treatment or control group and receive the according study medication, which will be consumed three times daily for the following 12 weeks. V2 takes approximately 60 min.

After 4 weeks of intervention, there will be another visit (V3, week 4), during which KB and glucose levels will be measured, adverse events will be recorded, vital signs, diet and migraine diary will be checked, a dose of IMP/placebo will be consumed, blood for safety will be drawn, physical examination will be performed if necessary, participants will be provided with the rest of the study medication for the first intervention period and sachets of used study medication will be collected for compliance control. V3 takes about 30 min.

During the visit after the first intervention period (V4, week 12), the following procedures are performed: migraine questionnaires, migraine diary and diet check, consumption of IMP/placebo, KB and glucose measurements, vital signs, blood draw for biomarker and safety analysis, physical examination if necessary and sachets of used study medication will be collected for compliance control. V4 takes about 60 min.

After 8 weeks without intervention, V5 (week 20) takes place, which is identical to the baseline visit (V2). V5 includes the following procedures: standardised migraine questionnaires, migraine diary and diet check, consumption of IMP/placebo, blood draw for biomarker and safety analysis, physical examination if necessary, KB and glucose concentration and vital signs. At this visit the patients will receive the alternative treatment to the first intervention.

After 4 weeks of the second intervention, V6 (week 24, analogous to V3) takes place. The following procedures are performed: migraine diary and diet check, consumption of IMP/placebo, KB and glucose measurements, adverse effects, vital signs, blood draw for safety and physical examination, if necessary. Participants will be provided with the rest of the study medicament and sachets of used study medication will be collected for compliance control. V5 takes about 30 min.

After completion of the second intervention, the last visit (V7, week 32, analogous to V4) takes place. The following procedures are performed: migraine questionnaires, migraine diary and diet check, consumption of IMP/placebo, KB and glucose measurements, vital signs and blood draw for biomarker and safety analysis, physical examination if necessary and collection of sachets of used study medication for compliance control.

All investigations will take place at the clinical trial unit of the University Hospital Basel, Switzerland. Participants are required to keep a detailed headache diary for the entire duration of the study.

### Sample size estimation

#### Determination of sample size

Sample size is estimated to be able to show the superiority of IMP over placebo. A crossover design with 1:1 IMP/placebo:placebo/IMP randomisation is planned.

#### Fixed sample size estimation

##### Assumptions

Sample size estimation is based on the following assumptions:

- We expect the baseline number of migraine days per 4 weeks to be 10 days in our patient population.
- Placebo effect: based on recent findings [64], we assume a rather strong placebo effect of 32% reduction in the primary endpoint. This corresponds to an absolute reduction of 3 migraine days per 4 weeks.
- IMP effect: synthesising previous findings [4, 64] and our pilot data [65], we aim to detect a difference of 2 days between placebo and IMP.
- We assumed the absolute reduction in migraine days to be normally distributed with a standard deviation of 3 days.
- We assume a conservative intra-patient correlation between IMP and placebo of 0.4.
- Drop out: a high drop-out rate of 30% is assumed.

##### Re-sampling

The sample size was estimated using a re-sampling method. Each sample size (n_i_ = 1,...,49 = 12, ..., 60) was evaluated by sampling *R* = 999 times the reduction in migraine days from a bivariate normal distribution as already described. For each sample, whether superiority of the IMP over placebo could be shown (i.e. whether a two-sided paired *t* test resulted in significant *p* < 0.05) was tested.

**Fig. 3:**
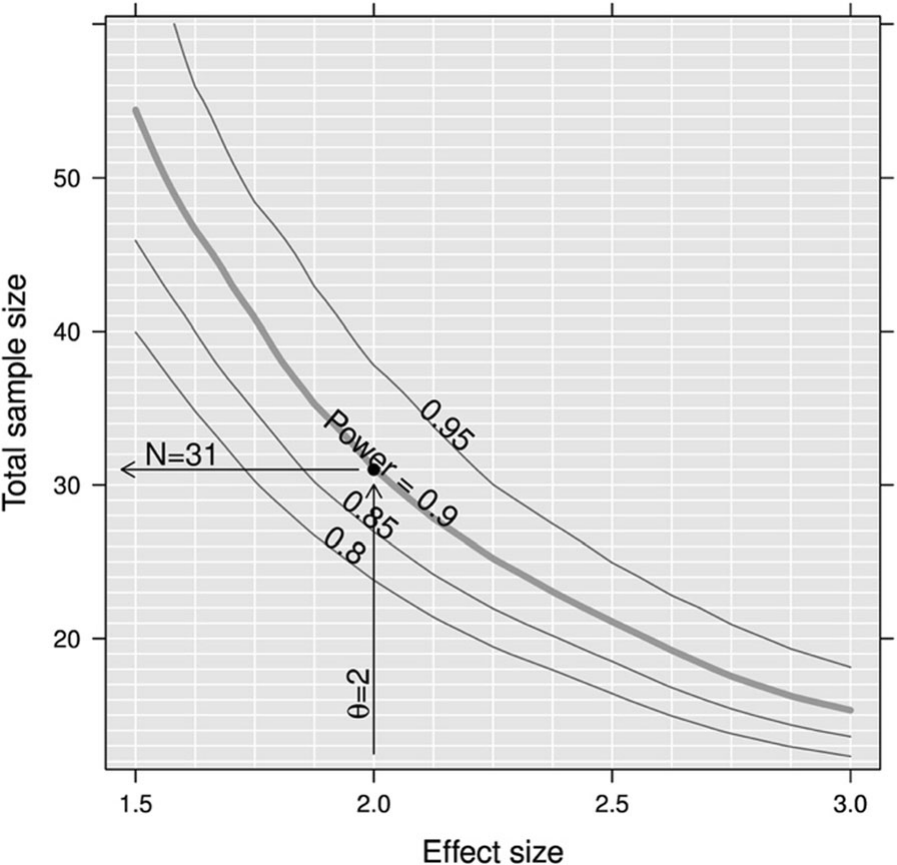
Sensitivity of sample size with regard to expected difference in reduction in number of migraine days per 4 weeks of IMP compared to placebo. Example given, based on an effect size of 2 and a statistical power of 90%. The curves are smoothed and are for illustrative purposes only

In order to show the superiority of the IMP over placebo with a statistical power of 90%, 45 patients should be recruited in total to ensure 31 evaluable patients, assuming a drop-out rate of 30%. Figure 3 shows how the sample size depends on the expected reduction in number of migraine days in the IMP arm.

### Recruitment

Patients will be informed about the study at the Department of Neurology, University Hospital Basel (USB). Moreover, there will by flyers publicly displayed in the waiting room of the neurology and general medicine department of the University Hospitals in Basel, Bern, Zurich and St Gallen, as well as the University Library. An announcement similar to the flyer will be posted on the webpages of the University of Basel “Marktplatz” dedicated to research studies (https://markt.unibas.ch/nc/inserate/kategorie/job-angebot-studien/) as well as the USB website (https://www.unispital-basel.ch/lehre-forschung/studieninserate/) and the University Children’s Hospital Basel (UKBB) website (http://www.ukbb.ch/en/research/research-groups/neuromuscular-research.php), respectively. More flyers will be displayed in local pharmacies and pharmacies in Germany (with a radius of approximately 100 km around Basel), local neurologists, the neurological department of the Bruderholzspital (Kantonsspital Baselland) and the Headache Clinic of RehaClinic, located in Baden as well as Bad Zurzach, and also in the neurological outpatient clinic in Brugg (team of Prof. Sandor). Flyers will also be displayed in local busses and trains. The Swiss Headache Society (SKG) and the German migraine and headache society (DMKG) will advertise the trial on their website. All websites may include the link to a short recruitment video (https://www.youtube.com/watch?v=2YzNjIXk_eY&t=19s) explaining the clinical trial with similar wording to the flyer. The video and an advertisement with similar wording to the flyer will also be advertised on Facebook (for users in a radius of 200 km of Basel). Patients previously contacted for a migraine–sport intervention study at the USB (EKNZ-Number 194/13) will be contacted again, if they previously agreed and met the inclusion criteria for the current study.

### Randomisation and blinding

#### Methods of minimising bias

Bias will be minimised by randomisation in 1:1 allocation and blinding of patients and investigators to the intervention. Randomisation will be done using an electronic data capture (EDC) system (SecuTrial) through an independent individual. The medication will be numerically labelled at the Pharmacy of the University Hospital of Basel and will then be provided to the ward and applied to the patient. This will allow a double-blinded randomisation (patient and treating physician will be blinded to the treatment).

The placebo powder has the same texture, colour and packaging as the IMP, so they cannot be distinguished in their appearance. The placebo also has a similar side-effect profile to the IMP. Data will be checked for protocol violation by the independent monitoring institution (see Quality assurance and control).

#### Randomisation

A crossover design with 1:1 AB/BA (IMP/placebo:placebo/IMP) randomisation is planned. The randomisation list will be computer generated and uploaded into the electronic data capture software SecuTrial by the responsible Data Manager at the Clinical Trial Unit (CTU) of the University Hospital Basel. Only unblinded personnel at the Pharmacy of the University Hospital Basel and at the CTU Basel will have access to the randomisation list. Just before the baseline visit, a clinical investigator will use SecuTrial to automatically assign a randomisation number from the randomisation list to the patient.

An additional list with medication numbers complementing the treatment arm of the first intervention period will be provided by the CTU, in order to allow opposite treatment allocation during the second intervention period without unblinding the trial staff.

#### Blinding procedures

The study medication (IMP or placebo) will be provided as similar-looking medication in sachets. The medication will be packed by the Pharmacy of the University Hospital of Basel and will be numerically labelled using the randomisation list provided by the responsible Data Manager at CTU Basel. All investigators and patients will remain blinded until the trial is completed and the database has been locked.

#### Unblinding procedures (code break)

In the case of problems and safety concerns that cannot be solved with ongoing randomisation, the participant’s allocated intervention will be revealed. Unblinding can be performed by authorised investigators using the EDC software SecuTrial. Each unblinding is documented in the EDC’s integrated audit trail system and automatically reported to the principal investigator.

### Data management

The study data recorded in the CRF will be transferred to a corresponding electronic CRF (e-CRF) by the clinical investigators. The principal investigator and co-investigator at the study site will be responsible for assuring that the data entered into the e-CRF is complete and accurate, and that the entry and updates are performed in timely manner. All information recorded in the e-CRFs will be traceable to the source documents in the patient’s file and in the date source files.

#### Data management system

Data management will be conducted fulfilling all ethical and legal requirements according to Good Clinical Practice (GCP) and the Swiss Laws as “Bundesgesetz über die Forschung am Menschen” (Humanforschungsgesetz (HFG)).

The e-CRF will be implemented by the data management group at the Clinical Trial Unit (CTU) of the University Hospital Basel using the electronic data capture (EDC) software SecuTrial. The EDC system runs on a server maintained by the IT department of the University Hospital Basel.

Data entry will be performed by trained clinical investigators at the UKBB.

#### Data security, access and back-up

The EDC system is accessible via a standard browser on a www-connected device. Password protection and user-right management ensures that only authorised UKBB or CTU staff can enter the system to view, add or edit data according to their permissions. User administration and user training is performed by the CTU Basel according to predefined processes.

Back-up of SecuTrial study data is performed regularly according to the processes of the IT department of the University Hospital Basel. An integrated audit trail system will maintain a record of initial entries and changes made, reasons for change, time and date of entry, and user name of the person authorising entry or change.

Source data will be available at the site to document the existence of the study participants and will include the original documents relating to the study (patient demographics, medical history, medication, neurological examination, informed consent forms).

#### Analysis and archiving

The EDC system will be locked after e-CRF data entry is completed, all data have been monitored and raised queries have been resolved. The complete study dataset is exported from the database and transferred to the study statistician as well as the principal investigator through a secure channel. The exported data will be archived for 10 years by the principal investigator.

#### Electronic and central data validation

Data entered into the e-CRF will be validated for completeness and discrepancies automatically. The data will be reviewed by the responsible investigator as well as an independent monitor. The monitor will raise queries using the query management system implemented in SecuTrial. Designated investigators have to respond to the query and confirm or correct the corresponding data. Thereafter, the monitor can close the query.

#### Data monitoring

To ensure the quality of the study conduct and of the data, monitoring of the study is performed by organisations independent of the study (CTU, USB and Kammermann Monitoring Services GmbH). All inclusion and exclusion criteria are checked, and the monitor controls whether the data have been recorded correctly in the CRF, whether the drug accountability is correct and whether serious adverse events (SAEs) have occurred during the study.

### Statistical analyses

Detailed methodology for summaries and statistical analyses of the data collected in this study will be documented in a statistical analysis plan. The statistical analysis plan will be finalised before database closure and will be under version control at the CTU, University Hospital Basel.

The primary endpoint, the number of migraine days in the last 4 weeks of treatment, will be measured twice for each patient, once after the placebo treatment period and once after the IMP treatment period. The number of migraine days in the 4 weeks before the start of treatment will be assessed for both treatment periods, thus there will be two baseline values that will be used as covariates. This process has the aim of correcting for any potential seasonal variation in baseline migraine frequency or carry-over effects.

#### Hypothesis

The *null hypothesis* is that there is no difference in the difference in number of migraine days per 4 weeks from baseline to the last 4 weeks of intervention between the IMP and the placebo treatment.

The corresponding *alternative hypothesis* is that the difference in the number of migraine days per 4 weeks from baseline to the last 4 weeks of intervention differs between the IMP and the placebo treatment.

#### Statistical criteria for termination of trial

No early stopping is planned, either for efficacy or for futility.

#### Planned analyses

##### Datasets to be analysed, analysis populations

The full analysis set (FAS) consists of all patients who are randomised and for whom the number of migraine days per 4 weeks at baseline is available.

The intention to treat (ITT) will include all randomised patients for whom the number of migraine days of at least the first 4 weeks of the first treatment period is available.

The per protocol (PP) will include all patients from the ITT set for whom the primary endpoint is available for both treatment periods, who are compliant as per the protocol (see later) and who have no protocol violations (to be defined in detail in the statistical analysis plan).

##### Primary analysis

The primary endpoint, the number of migraine days in the last 4 weeks of treatment, will be measured twice for each patient, once after the placebo treatment period and once after the IMP treatment period. The number of migraine days in the 4 weeks before start of treatment will be assessed for both treatment periods, thus there will be two baseline values that will be used as covariates. This process has the aim of correcting for any potential seasonal variation in baseline migraine frequency or carry-over effects.

The primary analysis will be performed using a linear, mixed-effects regression model. The primary model will include the primary endpoint (the number of migraine days in the last 4 weeks of treatment) as the response variable, the respective baseline value as a covariate, treatment (IMP vs placebo) and period (first vs second) as main effects, the two interaction terms “treatment × period” and “treatment × baseline value”, and patient as random effects. A significant interaction term between treatment and period would indicate a carry-over effect. Since it is not known how strongly the primary endpoint correlates with the baseline value, it is not known whether including the baselines as covariates in the model is sensible. Therefore, the already described primary model will be compared to models without the interaction term “treatment × baseline value” and without both the interaction term “treatment × baseline value” and the baseline value as a covariate by means of Akaike’s Information Criterion (AIC).

The primary analysis will be done on the ITT set.

##### Subgroup analyses

The following a priori defined subgroups will be investigated: sex (male/female), migraine with aura (yes/no) and baseline frequency of migraine days (medium = 5–9 days/4 weeks; high = 10–14 days/4 weeks). For each subgroup, the main effect of the subgroup and the interaction term “subgroup × treatment” will be added to the already described statistical model. In the case of a trend (*p* < 0.10) for an interaction effect—indicating a difference in the treatment effect between the subgroups—separate models will be fit for each subgroup.

##### Sensitivity analysis

The main analysis, without subgroup analyses, will be repeated on the PP set. Potential deviations from the results of the ITT analysis will be described in detail.

##### Secondary analysis

The secondary (exploratory) objectives are to assess the therapeutic efficacy of externally induced mild ketosis by the IMP regarding the following secondary endpoints:

- change in number of headache days of any severity from baseline (meeting ICHD-3 criteria) during the last 4 weeks of intervention;
- change in number of headache days of any severity from baseline (meeting ICHD-3 criteria) during the last 4 weeks of follow-up;
- change in consumption of acute migraine medication from baseline (analgesics or triptans)— measured in days with acute headache medication use—during the last 4 weeks of intervention;
- change in average migraine intensity from baseline—assessed with a VAS from 0 to 10 for each migraine episode—during the last of 4 weeks of the intervention period; and
- change in disability from baseline—assessed with the Migraine Disability Assessment (MIDAS) and the Headache Impact Test (HIT-6)—to the last of 4 weeks of the intervention period.

All of these secondary endpoints will be analysed as described for the primary endpoint with the corresponding baseline measure as covariate, if available.

All secondary analyses are done on the ITT set.

##### Exploratory analyses

The exploratory objectives are to assess the potential mechanisms of action of externally induced mild ketosis by the IMP regarding markers of oxidative stress, markers of inflammation, glucose, fat, protein metabolism and genetic analyses:

- Serum concentration changes from baseline of oxidative and nitrosative stress markers (malondialdehyde (MDA), carbonylated proteins, nitrate, nitrite, nitrotyrosine) using ELISA and mass spectroscopy. This exploratory endpoint will be analysed as described for the primary endpoint with the corresponding baseline measure as a covariate.
- Serum concentration changes from baseline in markers of fat (triglycerides, cholesterol, HDL, LDL) or glucose metabolism (insulin, glucose, cortisol, Hba1c and lactate) during the last 4 weeks of intervention. This exploratory endpoint will be analysed as described for the primary endpoint with the corresponding baseline measure as a covariate.
- Serum concentration changes from baseline in serum inflammatory markers (cytokines including, but not limited to, IFN-γ, IL-1β, IL-2, IL-4, IL-5, IL-6, IL-10, MCP-1, TNF-α, TNF-β, TGF-β1) during the last 4 weeks of intervention, using a multiplex immunoassay analysed with a BioPlex 200. This exploratory endpoint will be analysed as described for the primary endpoint with the corresponding baseline measure as a covariate.

The following exploratory endpoints will be analysed with standard methods for gene and/or gene expression variation analysis:

- genetic profile (single nucleotide polymorphisms (SNPs)) of all patients involved in the study and correlation of the genetic markers with other outcome measures;
- gene expression changes before and after diet using expression microarrays with a special focus on mitochondrial-related genes (citrate synthase, cytochrome C oxidase subunit 1, succinate dehydrogenase subunit A); and
- correlation of gene expression changes with the genetic profile of the patients (eQTL analysis in combination.

All exploratory analysis is done on the ITT set.

##### Safety analysis

Safety and tolerability will be determined by:

- comparison of treatment-emergent adverse events (any event regardless of potential causality with the drug) and treatment-related adverse events as defined by the principal investigator between active treatment and placebo; and
- examination for potential effects of the intervention on routine laboratory parameters (renal and liver function tests, electrolytes, full blood count, CRP, lipids, Hba1c, insulin, cortisol, lactate, TSH, FT4, FT3) in the treatment group compared to the control group.

##### Deviation(s) from the original statistical plan

If substantial deviations of the analysis as outlined in these sections are needed for whatever reason, the protocol will be amended. All deviations of the analysis from the protocol or from the detailed analysis plan will be listed and justified in a separate section of the final statistical report.

#### Handling of missing data and drop-outs

The frequency of, timing of and reasons for, as well as all side-effects of, drop-outs will be reported for each treatment. Patients who drop out during the first run-in period or during the first 4 weeks of the first treatment period will be excluded. All patients who drop out later will be included in the ITT set.

For patients who drop out after the first 4 weeks and before the end of the first treatment period, the primary endpoint for the first treatment period will be imputed using multiple imputations. If appropriate, imputations will be accounted for baseline value and number of migraine days during the first treatment period, as far as available. The primary endpoint for the second treatment period will not be imputed for these patients.

For patients who drop out after the end of the first treatment period and before the first 4 weeks of the second treatment period are finished, the primary endpoint for the second treatment period will not be imputed. The primary endpoint for the first treatment period will be available.

For patients who drop out after the first 4 weeks and before the end of the second treatment period, the primary endpoint for the second treatment period will be imputed as already described.

Thus, for each patient included in the ITT set, the primary endpoint will be available (whether measured or imputed) for at least the first treatment period and will be taken into account with the proposed mixed effects models.

In case there are indications for missing data not at random, the inverse probability of censoring weights (IPCW) will be considered.

##### Statistical criteria for termination of trial

No early stopping is planned, either for efficacy or for futility.

### Quality assurance and control

The principle investigator (PI) is responsible for implementing and maintaining quality assurance and quality control systems with written SOPs and Working Instructions. The PI is responsible for proper training of all involved study personnel. To assess high-quality conduct of the trial in accordance with the protocol, all medical staff involved in this study are certified in good clinical practice (GCP).

#### Data handling and record-keeping/archiving

Paper documents including the results of the blood analysis, the headache diaries, questionnaires and all study-related documents will be filed in the study files and stored in the hardcopy archive of UKBB on a dedicated shelf.

#### Case report forms

For each subject included in this study, a case report form (CRF) will be completed, dated and signed by a study investigator. Data will be recorded in the CRF from the source documents, which may include medical notes and results obtained from laboratory reporting systems.

All participants receive a unique identification number (patient ID) and no identifying data such as name, initials or birth date will be collected in the CRF.

#### Specification of source documents

Source data will be available at the site to document the existence of the study participants. Source data will include the original documents relating to the study (patient demographics, medical history, medication, neurological examination, informed consent forms) as well as the MIDAS and the HIT-6 questionnaire.

#### Record-keeping/archiving

All study data, including CRFs and informed consent forms, will be archived for a minimum of 10 years after termination (or premature termination) of the clinical research project. Paper documents including the results of the blood analysis and gene expression changes as well as questionnaires will be stored in the hardcopy archive of the UKBB.

#### Monitoring

To ensure the quality of the study conduct and of the data, monitoring of the study will be performed by a person independent of the study (Kammermann Monitoring Services GmbH, Zug, Switzerland). All inclusion and exclusion criteria will be checked and whether the data have been recorded correctly in the CRF, whether the drug accountability is correct and whether SAEs have occurred during the study.

#### Audits and inspections

All study documentation and the source data/documents will be accessible to auditors/inspectors (also EKNZ and CA) and questions will be answered during inspections. All involved parties must keep the participant data strictly confidential.

#### Confidentiality and data protection

Direct access to source documents will be permitted for purposes of audits and inspections (ICHE6, 6.10). The investigators of the study will have access to the protocol, dataset (including questionnaires, demographical/clinical data) and statistical code during and after the study. The patients’ identities will never be published in any abstracts or publications. A transfer of data will only take place for study purposes and only in encoded form. Third persons will not gain any insight into original data. For inspection purposes, insight into the original data will be permitted to the members of the appropriate authorities and also for the members of the local ethics committee, EKNZ. During the study, confidentiality will be guaranteed. The principal investigator will guarantee compliance with national and international data security.

#### Storage of biological material, related health data and returned study medication

Blood samples will be sent immediately to the earlier specified research laboratories. DNA and RNA extraction will be conducted immediately after arrival at the research laboratory. The extracted DNA/RNA will be sent for microarrays analysis on dry ice to Life&Brain, Bonn, Germany.

Biological material and related health data will be stored in an encrypted format for follow-up analyses.

In order to assess compliance of study medication intake, empty and full sachets are returned by the patients at visits 3, 4, 6 and 7. A member of the study team will count and balance the returned containers and can check the correct intake. This will be captured in an appropriate form. A qualified person from the study team will check the number of dispensed/taken medications and complete a study-specific drug accountability form. After completion of the clinical trial, leftover study medication will be destroyed.

### Safety assessments

Adverse events are monitored throughout the study. At every study visit, patients are asked about adverse events and their vital parameters are measured. If an AE is reported, a clinical examination is performed. The following safety parameters amongst other parameters are checked at visits 2, 3, 4, 5, 6 and 7 to determine safety of the treatment: routine laboratory parameters (renal and liver function tests, electrolytes, full blood count, C-reactive protein, serum cholesterol, triglycerides, serum proteins, albumin, glucose, Hba1c, insulin, cortisol, lactate, TSH, FT4 and FT3), blood pressure, heart rate, weight and height, assessed after 5 min of resting in a supine position.

As βHB is an endogenous substance we are not expecting any treatment-related serious adverse events on routine laboratory measures. Nevertheless, the intake of the IMPs will be stopped in the case of clinically significant changes in any of the parameters measured. In the event of any serious adverse events (treatment related or unrelated) occurring during intake of the IMPs, treatment will also be stopped immediately. If pathologic changes should be detected, whether related to or independent of migraine, the affected patients will be informed immediately and the possibilities of further investigation, respectively treatment of these abnormalities according to current medical knowledge, will be discussed.

#### Reporting of serious adverse events and other safety-related events

Treatment-emergent serious adverse events (any event regardless of potential causality with the drug) and treatment-related adverse events as imputed by the principal investigator (such as gastrointestinal upset) will be recorded. Reporting to the EKNZ will take place according to the clinical trials of medicinal products guidelines for notification and reporting of Swissethics. In brief:

- Serious adverse events (SAEs) with fatal consequences or where a connection is suspected with the intervention will be reported within 7 days.
- Suspected unexpected serious adverse reactions (SUSARs) with fatal consequences will be reported within 7 days, other SUSARs within 15 days.
- SAEs that may be related to the intervention under investigation in other clinical trials will be reported within 15 days.

AEs of this trial are graded in the most recent Common Terminology Criteria for Adverse Events (CTCAE) version 5.0, which was published in November 2017 and became effective in April 2018 [66], published by the National Cancer Institute (NCI) of the National Institutes of Health (NIH).

#### Follow-up of (serious) adverse events

Patients with adverse reactions which have occurred in the context of the study will be followed up by the investigator up to 30 days after the last visit.

## Discussion

We propose a single-centre, randomised, double-blind, placebo-controlled, crossover trial to determine whether treatment with βHB in mineral salt form has a positive effect on migraine frequency and associated symptoms. To our knowledge this is the first RCT using exogenous KB salts worldwide. If proven effective, βHB might offer a new prophylactic treatment option for moderately to strongly affected migraine patients, or at least a subgroup thereof. A demonstration of its safety might additionally pave the way for clinical trials assessing its use in related diseases.

Planning clinical trials in migraine is challenging for the following reasons: migraine is an episodic disease with a fluctuating nature (i.e. in some patients, migraine frequency can vary substantially from one month to the next or one season to the other, which makes it harder to demonstrate a treatment-related effect); the placebo effect is quite large, between 20 and 40% [67], which further adds to this problem; individual migraine attacks are of different length, and in more severely affected patients are sometimes hard to identify [68]; some patients suffer from headache of a different quality in addition to migraine and this distinction must be made by the patient subjectively [68]; and there is no objective biomarker for migraine or disease severity [68].

In order to address these problems, we have: incorporated two baseline periods to account for seasonal changes, and chosen a conservative effect size as well as a study population of moderate to high-frequency episodic migraineurs (5–14 headache days per month), in order to make it easier to demonstrate a sufficiently large effect size within a short timeframe, without introducing any confounds associated with chronic migraine, such as frequent co-morbidities [69]; calculated with a quite large placebo effect of 30%; chosen migraine days versus migraine attack frequency as the primary outcome; included a thorough briefing of each patient on the characteristic features of a migraine versus a headache attack; and included a detailed medical history and diagnostic consultation by a neurologist, as well as a carefully constructed headache diary.

We have decided in favour of a crossover design in this single-centre RCT for the following reasons. Despite all efforts, recruitment has been slow and screening failures were a little higher than expected; in addition, we found that patients tended to be discouraged when they learned that they had solely a 50% chance of trying the IMP and would only find out which treatment arm they belonged to upon trial completion (in over 2 years time).

A crossover design in migraine is typically not recommended [68] because of the following limitations [67]: the possibility of a carry-over effect; the need for a long total period of treatment (extended by a wash-out period) with concomitant increases in drop-outs over time and in turn loss of statistical power [70]; and the increased likelihood of adverse events, which can unmask the blinding when a subject is exposed to both treatments.

In our case, a crossover design has three key advantages:

1. A crossover design greatly improves statistical power, as each patient can be his/her own control (within-subject analysis versus between-subject analysis), which can be especially useful in a heterogeneous disease such as migraine, and hence fewer patients would be necessary to demonstrate a given effect. The sample size is effectively halved, even when more conservative a priori assumptions are employed, which is advantageous in single-centre studies. To compensate for some of the aforementioned weaknesses of crossover designs in migraine, we decided to make our a priori assumptions to determine the sample size more conservative than we would have with a parallel group design: a statistical power of 90% and a drop-out rate of 30% were chosen (in addition to a 30% placebo effect).
2. A crossover design gives each patient the chance to try the IMP, which—from our experience—increases compliance, motivation and participation rates. We asked 25 prospective subjects for their preference and all of them favoured a crossover over a parallel group design. Instead of 6 months including a follow-up period, patients are now participating for a total of 9 months. The longer duration might lead to a slight increase in drop-out rates; however, on the other hand, it also leads to much improved participation rates, while only needing half of the patients. Additionally, it is known that vigilant patient education, monitoring and follow-up may reduce drop-out rates in longer trails [70]. From our experience, the moderate increase in trial duration has nowhere near negatively outweighed the positive impact of being guaranteed exposure to the IMP. A subsequent open-label period at the termination of the parallel group design would have a similar effect, but would also increase the costs substantially, as it does not have any impact on statistical power. This can be problematic, particularly for investigator-initiated trials.
3. In addition to adding a wash-out period, the crossover design also allowed us to incorporate a second baseline period. This might help control for any potential seasonal effects on migraine frequency.

The possibility of a carry-over effect is always there; however, with a very short half-life of approximately 3–4 h, a 4-week wash-out period was judged to be sufficient.

Finally, we addressed the possibility of unblinding due to exposure to both substances. While there is no way to completely avoid this issue, we chose a placebo with a similar gastrointestinal side-effect profile to the IMP: mannitol, a sugar alcohol, can cause gastrointestinal disturbances, without having any systemic effect as it does not leave the gastrointestinal tract [58].

Various explorative outcomes have been included in order to be able to identify some of the potential protective mechanisms of exogenously induced ketosis in migraine. In addition, we are hoping this might help us distinguish responders and non-responders on both a phenotypical as well as physiological level.

## Trial status

The trial started enrolment in May 2017 and is expected to be completed by the end of January 2020.

The newest protocol version is V6 of 5 September 2018. All protocol modifications have been and will be reported to the local ethic committee (Swissethics) and other relevant parties (such as Swissmedics, investigators and trial participants).

## Additional file

Additional file 1: SPIRIT 2013 checklist: recommended items to address in a clinical trial protocol and related documents. (DOC 122 kb)

## Abbreviations

βHB: Beta-hydroxybutyrate
CA: Competent authority
Ca^2+^: Calcium ion
CRF: Case report form
CTU: Clinical Trial Unit, University Hospital Basel, University of Basel
EDC: Electronic data capture
EKNZ: Local ethic committee of northern central Switzerland
ELISA: Enzyme-linked immunosorbent assay
FAG: Freie Akademische Gesellschaft
FAS: Full analysis set
GCP: Good clinical practice
HFG: Humanforschungsgesetz
HIT-6: Headache Impact Test
ICHD-3: International Classification of Headache Disorders version 3
IMP: Investigational medicinal product
ITT: Intention to treat
KB: Ketone body
KD: Ketogenic diet
MDA: Malondialdehyde
Mg^2+^: Magnesium ion
MIDAS: Migraine Disability Assessment
MoH: Medication overuse headache
NSAID: Non-steroidal anti-inflammatory drug
PP: Per protocol
RCT: Randomised controlled trial
SAE: Serious adverse event
SKG: Swiss Headache Society
SNF: Swiss National Foundation
SNP: Single nucleotide polymorphism
SOP: Standard operating procedure
SUSAR: Suspected unexpected serious adverse reactions
UKBB: University Children’s Hospital Basel
USB: University Hospital Basel
V1: Visit 1
VAS: Visual analogue scale
